# An international comparison of diagnostic and management strategies for vestibular schwannoma

**DOI:** 10.1007/s00405-018-5199-6

**Published:** 2018-11-12

**Authors:** Mayke Hentschel, Maroeska Rovers, Laura Markodimitraki, Stefan Steens, Henricus Kunst

**Affiliations:** 1 Department of Otolaryngology, Radboud Institute for Health Sciences, Radboud University Medical Center, P.O. Box 9101, 6500 HB Nijmegen (377), The Netherlands; 2 Department of Operating Rooms, Radboud Institute for Health Sciences, Radboud University Medical Center, Nijmegen, The Netherlands; 3 Department of Health Evidence, Radboud Institute for Health Sciences, Radboud University Medical Center, Nijmegen, The Netherlands; 4 Department of Radiology & Nuclear Medicine, Radboud University Medical Center, Nijmegen, The Netherlands

**Keywords:** Vestibular schwannoma, Diagnosis, Management, Questionnaire, Otolaryngologists, International

## Abstract

**Objective:**

To compare international diagnostic and management strategies for vestibular schwannoma (VS).

**Methods:**

A web-based questionnaire was sent to 130 otolaryngologists, mainly identified through the European Skull Base Society. It contained questions on general information including guideline usage as well as questions on diagnosis (focussing on selection of patients for MRI) and management of VS, including case scenarios. Descriptive statistics were reported.

**Results:**

Thirty-six otolaryngologists working in 11 different countries completed the questionnaire (response rate: 28%). Guidelines for diagnosis and management of VS are used by 44% and 42% of respondents, respectively. In the diagnostic strategy for VS, different types and combinations of audiovestibular function tests are used when deciding whether a patient should undergo an MRI. Respondents apply 18 different definitions of asymmetrical hearing loss. Variation was also apparent from reported considerations on management of VS. Most respondents (84%) prefer a wait-and-scan strategy in case of a small intrameatal VS (Koos 1). Variety in management strategies increases for patients with a medium to large sized VS (Koos 2, 3 and 4). The details of each management strategy (wait-and-scan, microsurgery, stereotactic radiosurgery and fractionated radiotherapy) also differ among respondents.

**Conclusions:**

A large variation in diagnostic and management strategies for VS was identified between respondents. More evidence and/or consensus seem warranted to reduce uncertainties for patients, and differences in outcome and costs that might result from the variety of strategies currently being applied.

**Electronic supplementary material:**

The online version of this article (10.1007/s00405-018-5199-6) contains supplementary material, which is available to authorized users.

## Introduction

Patients with a vestibular schwannoma (VS) usually present with symptoms of (asymmetrical) sensorineural hearing loss, tinnitus, vertigo and/or disequilibrium. Magnetic resonance imaging (MRI) is considered the gold standard to diagnose VS and is performed whenever there is a high suspicion of VS in patients with aforementioned symptoms [1]. It is, however, a challenge to determine which patients should undergo MRI, the reported yield of diagnostic MRIs being approximately 3% [2, 3]. There are several tests available that can help to determine whether a patient should be referred for MRI. Pure tone audiometry is usually the first step in the diagnostic process and is sometimes followed by other audiovestibular function tests, such as auditory brainstem response, speech perception tests and electronystagmography [4, 5]. Although numerous studies examined the effectiveness of these audiovestibular function tests in selecting patients for MRI [1, 5–7], there seems no consensus regarding their role in everyday practice.

Apart from the variability in diagnostic strategies, there are multiple management strategies available for VS, consisting of microsurgical resection, radiation therapy (fractionated radiotherapy, stereotactic radiosurgery) or “wait-and-scan” (W&S, observation with serial imaging aiming to detect tumour growth). Over the past years the W&S strategy has gained popularity in Europe and the US [8, 9]. Treatment is increasingly being reserved for patients with a large size and/or growing VS. Because the natural growth pattern of VS is variable and unpredictable [10, 11], it is a challenge to determine the time interval between MRIs in the W&S strategy as well as indications for, and type of treatment.

An international guideline concerning the diagnosis and management of VS is lacking. Specialists seem to counsel their patients based on personal preference and experience [12]. A lack of guidelines prescribing the appropriate use of various strategies may contribute to inconsistencies in care delivery among specialists. These, in turn, may lead to uncertainties for patients, unnecessary differences in outcome between patients and unnecessary variation in costs associated with care. For these reasons, it is important to identify practice variations and possibilities to further improve healthcare.

In this study, we aimed to investigate variation in diagnostic and management strategies for VS across countries, and explore determinants of such variation.

## Methods

### Study design and population

To obtain information regarding the current diagnostic and management strategies for VS, an online questionnaire was sent to 102 otolaryngologists working in 15 different countries, that were registered in the European Skull Base Society database. We additionally invited 28 otolaryngologists whose contact details were acquired through hospital websites or by personal acquaintance (excluding the authors’ hospitals). The questionnaire was distributed using ‘Castor EDC’ [13] in January 2017. We sent two reminders in a time span of 2 weeks.

### Questionnaire

**Fig. 1:**
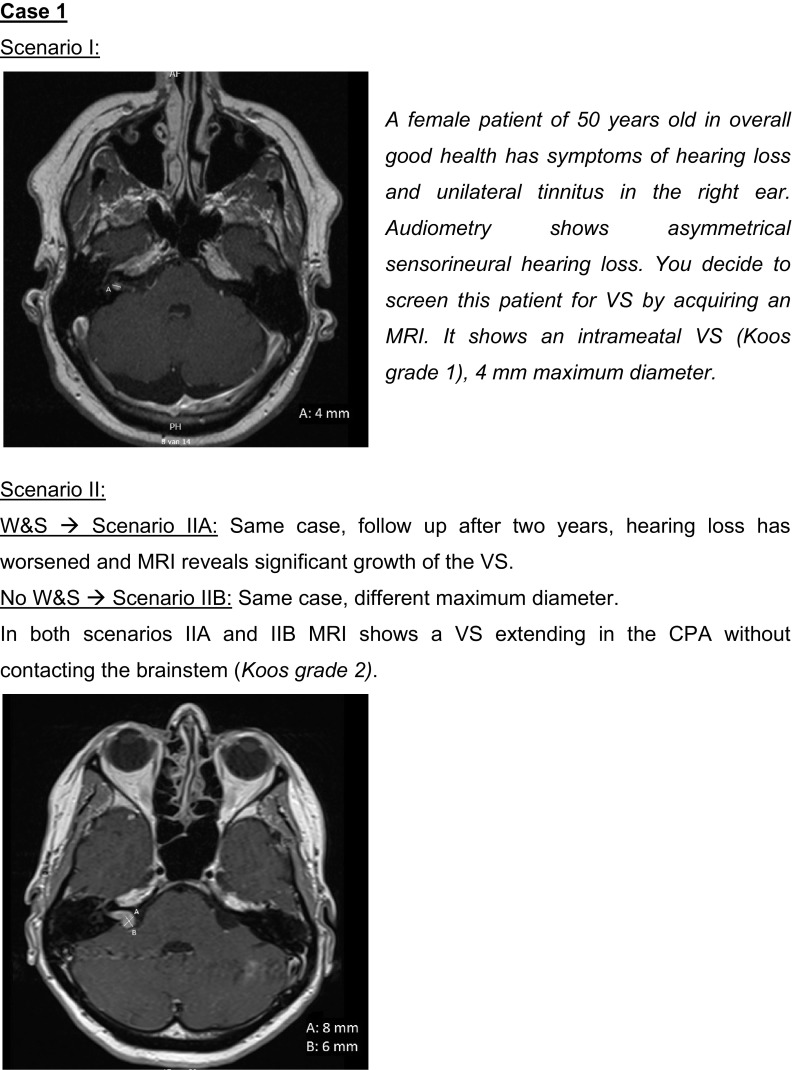

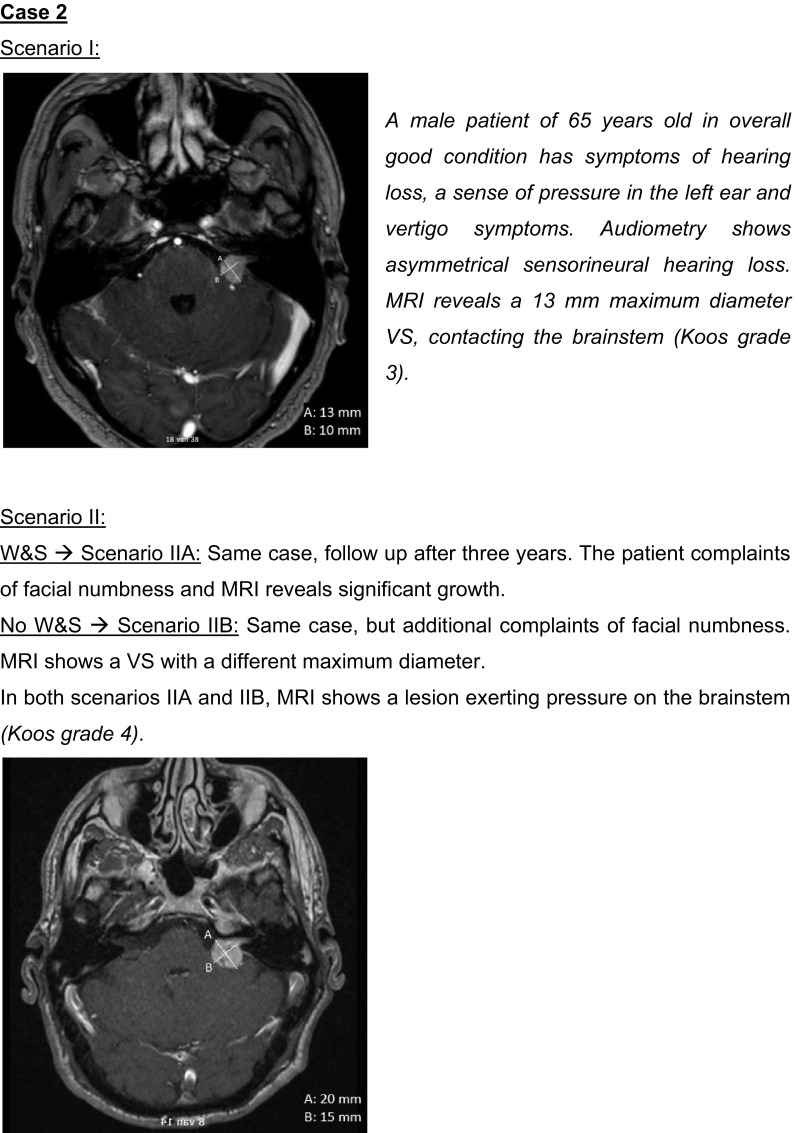
Case scenarios and MRI images used in the questionnaire

The questionnaire is provided as Supplemental file 1. It consisted of three main sections. The first section contained questions on general topics, e.g. patient volumes and guideline usage. The second section focused on audiovestibular function tests and investigated based on what parameters respondents refer a patient for MRI. In the third section different management strategies for VS were addressed, including the proportion of patients being assigned to each strategy, variables used when considering management strategies, and their conduct. It included several case scenarios describing VSs of increasing size (see Fig. 1). Respondents chose the preferred management option for each case, which enabled us to further explore their considerations and assess impact on individual patients. The questionnaire was tested prior to its distribution, and adapted accordingly to the comments received by two otolaryngologists, a professor in evidence based surgery, a radiologist specialized in neuro- and head and neck radiology, and a junior researcher in the field of VS.

### Data analysis

Diagnostic and management strategies were compared between respondents and countries, by providing descriptive statistics. Percentages and medians or means were calculated where applicable. Data were analyzed per item, so the denominator varied per question due to missing data. All analyses were performed using IBM SPSS Statistics for Windows, version 22 (IBM Corp., Armonk, N.Y., USA).

## Results

### Respondents

Of the 130 addressed, 36 otolaryngologists from 11 different countries returned the questionnaire (response rate 28%) (Supplemental table 1), visited by a median of 85 (*n* = 32, range 3–300) patients with newly diagnosed VSs in 2016. All respondents (*n* = 36) indicated to work at a university hospital and the mean experience in working with VS patients was 16 years (SD 7.3).

The majority of respondents (56%) never uses a guideline, neither for diagnosis nor management. Despite the availability of guidelines in the Netherlands (asymmetrical hearing loss and tinnitus) [14] and the United Kingdom (diagnosis and management) [9], heterogeneity was found in responses from these countries.

Thirty respondents (88%) participate in multidisciplinary meetings to discuss VS cases, while the remaining 4 (12%) do not have such meetings.

### Diagnostic strategies

All respondents have MRI available, and most (79%) use contrast-enhanced MRI to diagnose VS. There was more variety regarding the use of audiovestibular function tests to select patients for MRI. Most respondents (*n* = 31, 94%) use pure-tone audiometry in the diagnostic process, the remaining two respondents solely relying on auditory brainstem response. We investigated the definition of asymmetrical hearing loss that respondents apply when referring patients for MRI. The minimum asymmetry of hearing loss being used ranged from 5 to 30 dB, 20 dB being mostly used (*n* = 11, 36%). Most respondents (*n* = 27, 87%) define asymmetry as an absolute difference between ears at specific frequencies, while 4 (13%) calculate a mean. However, how many and which frequencies are taken into account as well as their mutual relation (adjacent or non-adjacent) varies. Respondents provided 18 different definitions of asymmetrical hearing loss, including 12 different combinations of frequencies that are considered important. The most frequently reported definition (*n* = 6, 19%) uses an absolute asymmetry of 10 dB as threshold, followed by an absolute asymmetry of 20 dB (*n* = 4, 13%).

Twenty-four respondents (73%) usually perform speech audiometry to decide if a patient should undergo MRI. Most (*n* = 14, 58%) use speech reception thresholds.

Moreover, respondents explained to look for the roll-over phenomenon as well as discrepancy with pure-tone audiometry measurements.

Twenty-one respondents (64%) order an MRI for patients with unilateral tinnitus as only symptom. Its duration varied (0–11 months), but most respondents apply a minimum of 3 months (*n* = 6, 29%). Twenty-one respondents (64%) perform electronystagmography in case of vertigo and 16 respondents (49%) use auditory brainstem response.

Additional audiovestibular function tests consisting of (video) head impulse tests and/or vestibular-evoked myogenic potentials are used by 4 and 3 respondents, respectively.

### Management strategies

Most respondents (*n* = 15, 42%) deem tumour size the most important variable when deciding about management strategies, followed by cerebellopontine angle size/intracranial space (*n* = 10, 28%). VS size is measured using dimensional and volumetric measurements by 27 (84%) and 5 (16%) respondents, respectively. Twenty respondents (63%) use a specific threshold in tumour size/volume when considering treatment, varying from 15 to 30 mm, a majority applying 20 mm (*n* = 9, 45%). Five respondents (16%) base their decision to proceed to treatment on tumour size/volume only, while most (*n* = 15, 47%) also consider other variables (e.g. brainstem contact and/or compression, patient characteristics and/or symptoms, and/or tumour growth).

**Fig. 2:**
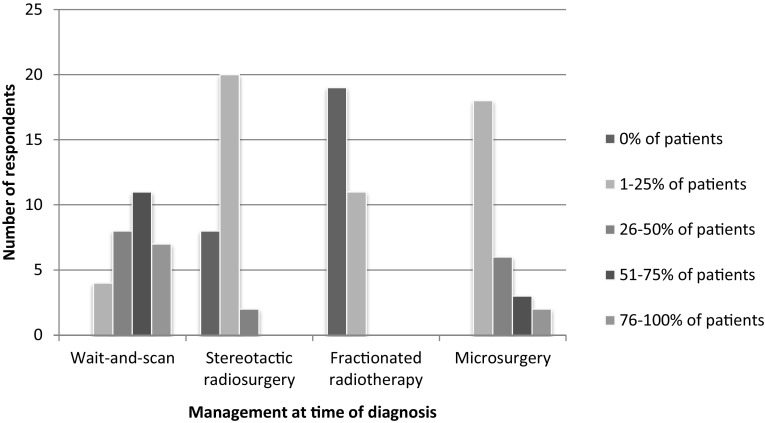
Fraction of VS patients assigned to management strategies at time of diagnosis, reported by 30 respondents. *y* axis: number of respondents, *x* axis: different management strategies at time of diagnosis

In 2016, the proportion of VS patients assigned to each management strategy at time of diagnosis varied (Fig. 2). Both microsurgery and W&S were applied in all participating centres in varying proportions. Twenty-one respondents (70%) assigned a majority of patients to W&S, while only 5 (17%) did so for microsurgery. Stereotactic radiosurgery was prescribed by more respondents (*n* = 22, 73%) than fractionated radiotherapy (*n* = 11, 37%).

### Wait-and-scan

**Fig. 3:**
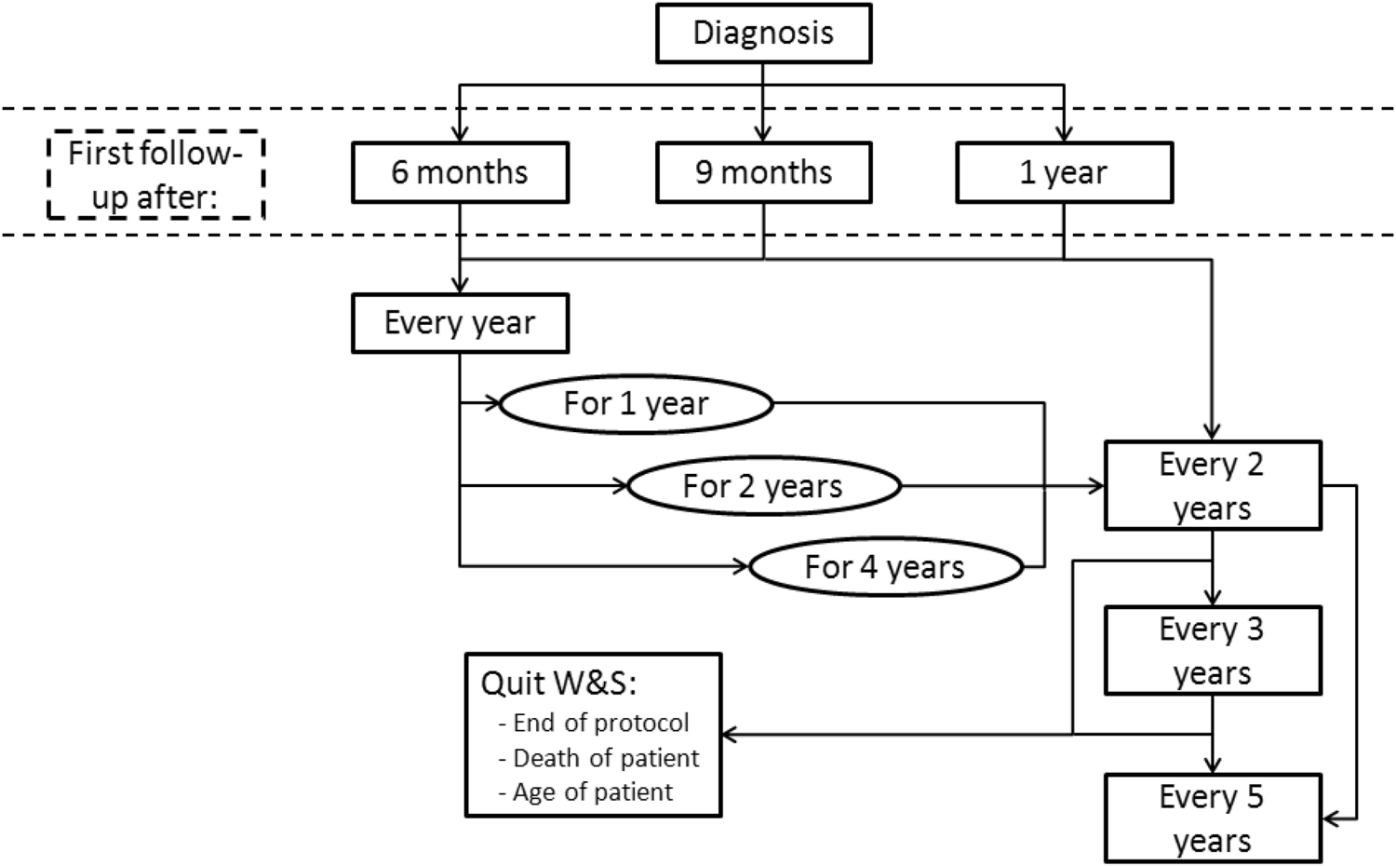
Display of variation in applied wait-and-scan (W&S) strategies

Figure 3 provides a schematic overview of W&S strategies being used. Strategies vary in timing of the first MRI following diagnosis, interval periods and total duration of the observational period. Respondents quit the W&S strategy when a patient reaches a specific age (75 and 80 years), after a specified period (4–21 years), or continue lifelong. Most (*n* = 18, 56%) define significant tumour growth as an increase in diameter of ≥ 2 mm, while others apply an increase of ≥ 1 mm (22%) or a volume increase of ≥ 10% (9%). Ten respondents (31%) consider tumour growth a strict indication for treatment, whereas 69% also take other variables into account (e.g. tumour size, symptoms and/or patient age, health and/or preference).

### Radiation therapy

Of the respondents prescribing stereotactic radiosurgery, most use Gamma Knife (*n* = 11, 52%). Fractionated radiotherapy treatment plans were reported by 10 respondents and contain up to 30 fractions, with total dosages of 12–60 Gy.

### Microsurgery

The microsurgical approach most frequently used by respondents is translabyrinthine (*n* = 20, 65%), followed by suboccipital (*n* = 6, 19%) and middle fossa (*n* = 5, 16%). Whenever the facial nerve is difficult to recognise intra-operatively, most respondents (*n* = 29, 94%) opt for incomplete VS removal to reduce the risk of facial nerve injury.

### Case scenarios

Table 1 displays the management strategies chosen for the different case scenarios (from Fig. 1). There is much agreement about conservative management for small intrameatal VSs, W&S being the most popular choice (84%) for Case 1—Scenario I. Even though all respondents indicated to allocate part of their patients to W&S, 5 (16%) preferred treatment over W&S for this scenario, motivated by the patient’s age (50 years) and chances of hearing preservation. For VSs extending in the CPA but not contacting the brainstem (Case 1—Scenario II) diversity in management choices becomes more apparent. For the VS that had grown during W&S (Case 1—Scenario IIA) some respondents would let the patient choose between W&S and treatment, and/or different treatment modalities. For the VS making brainstem contact (Case 2—Scenario I), preferred management options consisted mostly of W&S or microsurgery. For the largest VS causing brainstem compression (Case 2—Scenario II) differences were reduced again, microsurgery being most popular. The case scenarios confirm that fractionated radiotherapy is an uncommon treatment option for VS. Remarkably, some respondents working in the same centre chose different management options for the same case scenario. It seemed that otolaryngologists from high volume centers (> 100 new VS patients per year) preferred W&S over treatment for Case 2—Scenario I. For the other case scenarios we could not identify a difference between low and high volume centers.

**Table 1:** Management strategies chosen for two patients with different clinical characteristics

Management strategy	Case 1			Case 2		
	Scenario I *N* = 32 [*n* (%)]	Scenario IIA *n* = 27^a^ [*n* (%)]	Scenario IIB *n* = 5^b^ [*n* (%)]	Scenario I *N* = 32 [*n* (%)]	Scenario IIA *n* = 16^a^ [*n* (%)]	Scenario IIB *n* = 16^b,c^ [*n* (%)]
W&S	27 (84)	4 (15)	–	16 (50)	1 (6)	–
RS	–	11 (41)	–	1 (3)	4 (25)	–
RT	1 (3)	–	–	1 (3)	1 (6)	–
MS	4 (13)	12 (44)	5 (100)	14 (44)	10 (63)	14 (88)

Scenarios were adapted to choices made in scenario I

*W&S* wait-and-scan, *RS* stereotactic radiosurgery, *RT* fractionated radiotherapy, *MS* microsurgery

^a^Respondents that had chosen a W&S strategy in scenario I

^b^Respondents that had chosen to proceed to treatment in scenario I

^c^Two respondents that completed Scenario I did not complete this question

### Between-country comparisons

In the six countries with multiple responders, we identified a lot of heterogeneity making a comparison difficult. Some consistency was found in the UK, where most respondents use unenhanced MRI and all consider unilateral tinnitus (of various durations) as only symptom an indication for MRI. Respondents that mostly use the middle fossa approach work in Germany. Based on the case scenarios it seems that respondents working in Germany, the USA and France more often proceed to microsurgery compared to other countries.

## Discussion

Our questionnaire regarding diagnostic and management strategies for VS identified and explored variations in clinical practice. Less than half of respondents use a guideline for diagnosis and management. Respondents apply many different strategies to select patients for MRI and apply different thresholds for treatment. The case scenarios emphasized the impact this has on choices for VS management in individual patients.

Our results are in agreement with a study that reported on variations in disease presentation and initial management of small to medium sized VSs in the USA [12]. The study described place of residence as a stronger predictor for choice of management strategy than a patient’s age or VS size [12]. Next to referral patterns and availability of care, the authors contribute this to provider or institutional preference [12]. Naturally, in the current study, the proportion of patients assigned to each management strategy can be partially attributed to differences in local availability of care and referral patterns. However, our study also points out the various thresholds that otolaryngologists apply for diagnosis and management of VS, which will not depend on latter factors.

To our knowledge this is the first study to provide an overview of international differences in diagnosis and management of VS. The reliability of our data is dependent on accurate reporting of each otolaryngologist, which seems fair. However, selection of respondents cannot be precluded. Our results may, therefore, not comprise all strategies that are currently being used for diagnosis and management of VS. However, most included respondents work in high-volume centres and are responsible for consulting a substantial amount of VS patients. Furthermore, the variation might only further increase rather than becoming less after including more otolaryngologists. Preferably we would have achieved a higher response rate. Some non-responders motivated why they did not participate (i.e. retirement, no otolaryngologist, working in a private practice). Of the invited people, there was a larger proportion of professors that did not respond. Furthermore, we could not identify any differences between responders and non-responders. We deliberately limited our study population to otolaryngologists as we wanted to use one general questionnaire for both diagnosis and management of VS. Neurosurgeons compose an important link in the management of VS patients. However, otolaryngologists will be aware of the management of patients in their clinic/region, although they might not be treating them in person.

The lack of uniformity in diagnosis and management of VS is emphasized by the current study. The reported diagnostic work-up showed great variation, remarkably even by respondents from the same centre. The case scenarios revealed that this variation has an even greater impact on management strategies for individual patients than we had expected. Every otolaryngologist should realise that a patient might be managed differently elsewhere, even by a colleague within their own centre. We believe this information should not be neglected during patient counselling.

The current study cannot confirm whether different strategies also lead to differences in patient outcomes and costs. However, considering the extent of differences this does seem inevitable. The same patient might or might not be selected to undergo an MRI when visiting a different otolaryngologist. It is also, for example, known that facial nerve and hearing outcomes differ following radiation therapy and microsurgery [15]. It should be noted that experience with a certain treatment modality affects the results achieved. What sources of information underlie current strategies is largely unclear. Factors such as the training or reimbursements received by participants, participants’ age, access to diagnostic means and treatment and involvement in research projects might influence choices made. It seems that more evidence and/or consensus could reduce uncertainties for patients as well as potential differences in outcome and associated costs. Based on the currently available evidence, it is difficult to state what diagnostic strategy should be followed [4]. Moreover, there is a lack of evidence (functional outcomes and quality of life) on different treatment modalities stratified according to VS size. Combined with the current variety in applied strategies, this constitutes a challenge to implement an (inter)national guideline. Therefore, we encourage the exchange of evidence and considerations between otolaryngologists to try to reach consensus on a(n) (inter)national level.

## Conclusion

There is a high variability regarding diagnostic and management strategies for VS between otolaryngologists across countries. Otolaryngologists working in this field should realise these differences exist to optimize patient counselling. Further exploration of this variability may provide opportunities to synthesize the available evidence and to discuss possibilities to reach consensus.

## Electronic supplementary material

Below is the link to the electronic supplementary material.

Supplementary material 1 (DOCX 11 KB)

Supplementary material 2 (DOCX 170 KB)
