# Supplementary material for: An international comparison of diagnostic and management strategies for vestibular schwannoma
Source: Eur Arch Otorhinolaryngol. 2018 Nov 12;276(1):71–8. doi: 10.1007/s00405-018-5199-6 (PMC6338706; doi:10.1007/s00405-018-5199-6)
Supplement: Supplementary file 1 — Supplementary material 1 (DOCX 11 KB) [file 405_2018_5199_MOESM1_ESM.docx]

Supplemental table 1: Countries included in the study.

| Selected countries | Number of otolaryngologists | Number of responses |
| --- | --- | --- |
| Austria | 1 | - |
| Belgium | 3 | 3 |
| Denmark | 1 | 1 |
| France | 6 | 3 |
| Germany | 70 | 10 |
| Italy | 6 | 1 |
| New Zealand | 1 | 1 |
| Norway | 1 | - |
| Poland | 4 | 1 |
| Spain | 1 | - |
| Sweden | 3 | 1 |
| Switzerland | 1 | - |
| The Netherlands | 11 | 4 |
| United Kingdom | 11 | 7 |
| United States of America | 10 | 4 |
| Total | 130 | 36 |
