# Supplementary material for: An international comparison of diagnostic and management strategies for vestibular schwannoma
Source: Eur Arch Otorhinolaryngol. 2018 Nov 12;276(1):71–8. doi: 10.1007/s00405-018-5199-6 (PMC6338706; doi:10.1007/s00405-018-5199-6)
Supplement: Supplementary file 2 — Supplementary material 2 (DOCX 170 KB) [file 405_2018_5199_MOESM2_ESM.docx]

**Survey: Vestibular schwannoma guideline comparison**

The questionnaire consists of three parts, namely: general information, diagnosis and management.

- = one answer possible
- = multiple answers possible

The text in ***bold/italic*** indicates a dependency; the question will only appear if the stated text applies to previous answers given.

**General information**

The following questions will help us form a general idea about you and the centre you work.

| **Number** | **Question** | **Answers** |  |
| --- | --- | --- | --- |
|  | The following questions will help us form a general idea about you and the centre you work. | | |
| 1.1 | In which medical centre do you work? (please name centre, city and country) | \|  \| **Please fill in:** \| \| --- \| --- \| \| Centre \|  \| \| City \|  \| \| Country \|  \| |  |
| 1.2 | In what type of hospital/clinic do you work? |  University Hospital   Community Hospital   Other |  |
| 1.2.1 | ***If 'In what type of hospital/clinic do you work?' is equal to 'Other' answer this question:*** Please fill in the type of hospital/clinic you work. |  |  |
| 1.3 | For how many years have you been managing vestibular schwannomas? |  year(s) |  |
| 1.4 | Do you follow a guideline/protocol when consulting a patient with asymmetrical sensorineural hearing loss SUSPECTED OF vestibular schwannoma? |  Yes   No |  |
| 1.4.1 | ***If 'Do you follow a guideline/protocol when consulting a patient with asymmetrical sensorineural hearing loss SUSPECTED OF vestibular schwannoma?' is equal to 'Yes' answer this question:*** Please fill in the title of the protocol/guideline you follow. |  |  |
| 1.4.2 | ***If 'Do you follow a guideline/protocol when consulting a patient with asymmetrical sensorineural hearing loss SUSPECTED OF vestibular schwannoma?' is equal to 'Yes' answer this question:*** We would be pleased if you would share the above mentioned guideline with us. You can upload the file here. (optional) |  |  |
| 1.4.3 | ***If 'Do you follow a guideline/protocol when consulting a patient with asymmetrical sensorineural hearing loss SUSPECTED OF vestibular schwannoma?' is equal to 'Yes' answer this question:*** What kind of guideline/protocol is it? |  National   Regional   Hospital   Personal |  |
| 1.5 | Do you follow a guideline/protocol when consulting a patient DIAGNOSED WITH vestibular schwannoma? |  Yes   No |  |
| 1.5.1 | ***If 'Do you follow a guideline/protocol when consulting a patient DIAGNOSED WITH vestibular schwannoma?' is equal to 'Yes' answer this question:*** Please fill in the title of the protocol/guideline you follow. |  |  |
| 1.5.2 | ***If 'Do you follow a guideline/protocol when consulting a patient DIAGNOSED WITH vestibular schwannoma?' is equal to 'Yes' answer this question:*** We would be pleased if you would share the above mentioned guideline with us. You can upload the file here. (optional) |  |  |
| 1.5.3 | ***If 'Do you follow a guideline/protocol when consulting a patient DIAGNOSED WITH vestibular schwannoma?' is equal to 'Yes' answer this question:*** What kind of guideline/protocol is it? |  National   Regional   Hospital   Personal |  |
| 1.6 | Is there a workgroup or other meeting of some sort in your centre where vestibular schwannoma cases can be discussed? |  Yes   No |  |

**Diagnosis**

| **Number** | **Question** | **Answers** |  |
| --- | --- | --- | --- |
|  | The following questions regard the strategy you follow when consulting a patient suspected of vestibular schwannoma. | | |
| 2.1 | Which of the following NON-IMAGING tests do you USUALLY perform when suspecting a patient of vestibular schwannoma? |  Pure tone audiometry   Speech audiometry   Electronystamography   Auditory brainstem response   Other |  |
| 2.1.1 | ***If 'Which of the following NON-IMAGING tests do you USUALLY perform when suspecting a patient of vestibular schwannoma?' is equal to 'Other' answer this question:*** What is the non-imaging test that you usually perform when suspecting a patient of vestibular schwannoma? |  |  |
| 2.1.2 | ***If 'Which of the following NON-IMAGING tests do you USUALLY perform when suspecting a patient of vestibular schwannoma?' is equal to 'Pure tone audiometry' answer this question:*** When interpreting an audiogram of a patient suspect for vestibular schwannoma, how do you define asymmetry in the pure tone audiogram? |  I calculate an average value of frequencies   I look at absolute asymmetry (instead of averages) at  certain frequencies |  |
| 2.1.2.1 | ***If 'When interpreting an audiogram of a patient suspect for vestibular schwannoma, how do you define asymmetry in the pure tone audiogram?' is equal to 'I calculate an average value of frequencies' answer this question:*** How do you calculate the average pure tone audiometry value? |  I calculate the average pure tone audiometry values of  consecutive frequencies.   I calculate the average pure tone audiometry value of  non-consecutive frequencies.   Other |  |
| 2.1.2.1.1 | ***If 'How do you calculate the average pure tone audiometry value?' is equal to 'Other' answer this question:*** Please explain how you calculate the pure tone audiometry average. |  |  |
| 2.1.3 | ***If 'Which of the following NON-IMAGING tests do you USUALLY perform when suspecting a patient of vestibular schwannoma?' is equal to 'Pure tone audiometry' answer this question:*** Which of the following frequencies do you take into consideration when interpreting an audiogram of a patient suspected of vestibular schwannoma? |  500 Hz   1000 Hz   2000 Hz   3000 Hz   4000 Hz   6000 Hz   8000 Hz |  |
| 2.1.4 | ***If 'Which of the following NON-IMAGING tests do you USUALLY perform when suspecting a patient of vestibular schwannoma?' is equal to 'Pure tone audiometry' answer this question:*** What is the minimum asymmetry of hearing loss for which you would screen a patient? (dB difference between ears) |  dB |  |
| 2.2 | Here you can elaborate on your diagostic strategy. (optional) |  |  |
| 2.1.5 | ***If 'Which of the following NON-IMAGING tests do you USUALLY perform when suspecting a patient of vestibular schwannoma?' is equal to 'Speech audiometry' answer this question:*** Do you take the speech recognition score into consideration when deciding if a patient is a candidate for vestibular schwannoma screening? |  Yes   No |  |
| 2.1.5.1 | ***If 'Do you take the speech recognition score into consideration when deciding if a patient is a candidate for vestibular schwannoma screening?' is equal to 'Yes' answer this question:*** Please specify how the speech recognition score is taken into account. |  |  |
| 2.3 | Which of the following is your test of choice for diagnosing vestibular schwannoma? |  Magnetic Resonance Imaging   Auditory Brainstem Response   Other |  |
| 2.3.1 | ***If 'Which of the following is your test of choice for diagnosing vestibular schwannoma?' is equal to 'Other' answer this question:*** What is the diagnostic test of choice in your centre? Please avoid abbreviations when filling in. |  |  |
| 2.3.2 | ***If 'Which of the following is your test of choice for diagnosing vestibular schwannoma?' is equal to 'Magnetic Resonance Imaging' answer this question:*** Do you use contrast-enhanced (e.g. Gadolinium) MRI scan in all patients, when performing the DIAGNOSTIC scan? |  Yes   No |  |
| 2.4 | Do you perform an electronystamography when a patient reports vertigo symptoms? |  Yes   No |  |
| 2.5 | If a patient has unilateral tinnitus as the only symptom, do you screen for vestibular schwannoma? |  Yes   No |  |
| 2.5.1 | ***If 'If a patient has unilateral tinnitus as the only symptom, do you screen for vestibular schwannoma?' is equal to 'Yes' answer this question:*** What is the minimum duration of the unilateral tinnitus symptoms above which you screen for vestibular schwannoma? |  month(s) |  |
| 2.6 | Here you can elaborate on your diagnostic strategy. (optional) |  |  |

**Management**

| **Number** | **Question** | **Answers** |  |
| --- | --- | --- | --- |
|  | The following questions regard the management strategies of vestibular schwannoma in your centre. Please fill in all the questions. | | |
| 3.1 | Which of the following treatment options are either available in your centre or in a centre to which you refer your patients? |  Microsurgery   Radiosurgery   Radiotherapy |  |
| 3.2 | Approximately how many NEW patients with vestibular schwannoma are referred to your centre/hospital yearly? |  patients |  |
| 3.3 | IMMEDIATELY after diagnosis, what management strategy was chosen for your patients diagnosed with vestibular schwannoma in 2016? Please fill in estimated percentages so that they add up to 100%. | \|  \| **Please fill in the percentages** \| \| --- \| --- \| \| Wait and scan \|  \| \| RadioSURGERY \|  \| \| Microsurgical removal \|  \| \| RadioTHERAPY \|  \| |  |
| 3.4 | How important are the following factors when determining your management strategy IMMEDIATELY AFTER DIAGNOSIS of a vestibular schwannoma? Please fill in order of importance. If a factor is not applicable in your practice, you can leave the field blank.  (Choices: tumour size, cerebellopontine angle size/intracranial space, tumour consistency (solid/cyst), tumour localization, patient symptoms, age patient, overall health) | \|  \| **Factors in order of importance (from most important to least important)** \| \| --- \| --- \| \| Number 1 (MOST IMPORTANT) \|  \| \| Number 2 \|  \| \| Number 3 \|  \| \| Number 4 \|  \| \| Number 5 \|  \| \| Number 6 \|  \| \| Number 7 (LEAST IMPORTANT) \|  \| |  |
| 3.5 | How do you measure the size of a vestibular schwannoma on MRI? |  Dimensional measurement(s)   Volumetrics |  |
| 3.6 | When deciding between management strategies for vestibular schwannoma, do you take the remaining cerebellopontine angle space, in relation to the size of the tumour, into consideration? |  Yes   No |  |
|  | Radiosurgery | | |
| 3.1.2 | ***If 'Which of the following treatment options are either available in your centre or in a centre to which you refer your patients?' is equal to 'Radiosurgery' answer this question:*** What is the stereotactic radiosurgery of your choice? |  Gamma Knife   Linear Accelerator   Cyberknife   Other |  |
| 3.1.2.1 | ***If 'What is the stereotactic radiosurgery of your choice?' is equal to 'Other' answer this question:*** What stereotactic radiosurgery do you use? |  |  |
|  | Microsurgery | | |
| 3.1.4 | ***If 'Which of the following treatment options are either available in your centre or in a centre to which you refer your patients?' is equal to 'Microsurgery' answer this question:*** Which surgical approach do you use the most when performing microsurgery to remove a vestibular schwannoma? |  Suboccipital (including retrosigmoid)   Translabyrinthine   Middle fossa   No clear preference |  |
| 3.1.5 | ***If 'Which of the following treatment options are either available in your centre or in a centre to which you refer your patients?' is equal to 'Microsurgery' answer this question:*** If the facial nerve is difficult to recognise intraoperatively, do you choose radical removal of the vestibular schwannoma (with a higher risk of facial nerve dysfunction)? |  Yes   No |  |
| 3.1.6 | ***If 'Which of the following treatment options are either available in your centre or in a centre to which you refer your patients?' is equal to 'Microsurgery' answer this question:*** Please motivate your choice here. (optional) |  |  |
|  | Radiotherapy | | |
| 3.1.8 | ***If 'Which of the following treatment options are either available in your centre or in a centre to which you refer your patients?' is equal to 'Radiotherapy' answer this question:*** What is the fractionated radiotherapy treatment plan you use in your centre? | \|  \| **Please fill in below** \| \| --- \| --- \| \| Number of fractions \|  \| \| Dose per fraction (Gy) \|  \| \| Total dose (Gy) \|  \| |  |
| 3.1.9 | ***If 'Which of the following treatment options are either available in your centre or in a centre to which you refer your patients?' is equal to 'Radiotherapy' answer this question:*** Please elaborate further on your management strategy. (optional) |  |  |
|  | Wait and scan | | |
| 3.5.1 | ***If 'How do you measure the size of a vestibular schwannoma on MRI?' is equal to 'Dimensional measurement(s)' answer this question:*** Is 'wait and scan' a management strategy you use in your centre? |  Yes   No |  |
| 3.5.2 | ***If 'How do you measure the size of a vestibular schwannoma on MRI?' is equal to 'Volumetrics' answer this question:*** Is 'wait and scan' a management strategy you use in your centre? |  Yes   No |  |
| 3.5.1.1 | ***If 'Is ‘wait and scan’ a management strategy you use in your centre?' is equal to 'Yes' answer this question:*** When deciding between management strategies, is there a certain tumour size cut-off point above which you proceed to treatment? |  No, I don't use a cut-off point   Yes, but I also look at other variables besides tumour size   Yes, I always proceed to treatment above a certain tumour size |  |
| 3.5.1.1.1 | ***If 'When deciding between management strategies, is there a certain tumour size cut-off point above which you proceed to treatment?' is not equal to 'No, I don’t use a cut-off point' answer this question:*** What is the usual (minimum) cut-off point you use when deciding if a patient must undergo treatment? (please specify if you use millimeters or cubic millimeters) |  |  |
| 3.5.2.1 | ***If 'Is ‘wait and scan’ a management strategy you use in your centre?' is equal to 'Yes' answer this question:*** When deciding between management strategies, is there a certain tumour size cut-off point above which you proceed to treatment? | No, I don't use a cut-off point   Yes, but I also look at other variables besides tumour size   Yes, I always proceed to treatment above a certain tumour size |  |
| 3.5.2.1.1 | ***If 'When deciding between management strategies, is there a certain tumour size cut-off point above which you proceed to treatment?' is not equal to 'No, I don’t use a cut-off point' answer this question:*** What is the usual (minimum) cut-off point you use when deciding if a patient must undergo treatment? (please specify if you use millimeters or cubic millimeters) |  |  |
| 3.5.1.2 | ***If 'Is ‘wait and scan’ a management strategy you use in your centre?' is equal to 'Yes' answer this question:*** You can elaborate on the criteria for treatment here. (optional) |  |  |
| 3.5.2.2 | ***If 'Is ‘wait and scan’ a management strategy you use in your centre?' is equal to 'Yes' answer this question:*** You can elaborate on the criteria for treatment here. (optional) |  |  |
| 3.5.1.3 | ***If 'Is ‘wait and scan’ a management strategy you use in your centre?' is equal to 'Yes' answer this question:*** Which definition of (extrameatal) tumour growth do you use? Note that we mean growth from the moment of diagnosis. | Increase greater or equal to 0.5 mm   Increase greater or equal to 1.0 mm   Increase greater or equal to 2.0 mm   Other   Not applicable |  |
| 3.5.1.3.1 | ***If 'Which definition of (extrameatal) tumour growth do you use? Note that we mean growth from the moment of diagnosis.' is equal to 'Other' answer this question:*** Please fill in the definition of tumour growth that you use in your centre. | mm |  |
| 3.5.2.3 | ***If 'Is ‘wait and scan’ a management strategy you use in your centre?' is equal to 'Yes' answer this question:*** Which definition of (extrameatal) tumour growth do you use? Note that we mean growth from the moment of diagnosis. | Increase in tumour volume greater or equal to 10%   Increase in tumour volume greater or equal to 20%   Other   Not applicable |  |
| 3.5.2.3.1 | ***If 'Which definition of (extrameatal) tumour growth do you use? Note that we mean growth from the moment of diagnosis.' is equal to 'Other' answer this question:*** Please fill in the definition of tumour growth that you use in your centre. | mm3 |  |
| 3.5.1.4 | ***If 'Is ‘wait and scan’ a management strategy you use in your centre?' is equal to 'Yes' answer this question:*** Do you proceed to treatment when a tumour shows signs of growth? | Always   Sometimes   Never |  |
| 3.5.1.4.1 | ***If 'Do you proceed to treatment when a tumour shows signs of growth?' is not equal to 'Never' answer this question:*** Please elaborate on your answer. (optional) |  |  |
| 3.5.2.4 | ***If 'Is ‘wait and scan’ a management strategy you use in your centre?' is equal to 'Yes' answer this question:*** Do you proceed to treatment when a tumour shows signs of growth? | Always   Sometimes   Never |  |
| 3.5.2.4.1 | ***If 'Do you proceed to treatment when a tumour shows signs of growth?' is not equal to 'Never' answer this question:*** Please elaborate on your answer. (optional) |  |  |
| 3.5.1.5 | ***If 'Is ‘wait and scan’ a management strategy you use in your centre?' is equal to 'Yes' answer this question:*** What 'wait-and-scan' protocol do you use? Please fill in how often you scan a patient post diagnosis and for how many years. (e.g. year 1, 2, 3, 5, 7, 9, 15, 20) |  |  |
| 3.5.2.5 | ***If 'Is ‘wait and scan’ a management strategy you use in your centre?' is equal to 'Yes' answer this question:*** What 'wait-and-scan' protocol do you use? Please fill in how often you scan a patient post diagnosis and for how many years (e.g. year 1, 2, 3, 5, 7, 9, 15, 20). |  |  |
| 3.5.1.6 | ***If 'Is ‘wait and scan’ a management strategy you use in your centre?' is equal to 'Yes' answer this question:*** Please elaborate further on your wait and scan strategy. (optional) |  |  |
| 3.5.2.6 | ***If 'Is ‘wait and scan’ a management strategy you use in your centre?' is equal to 'Yes' answer this question:*** Please elaborate further on your wait and scan strategy. (optional) |  |  |
|  | Case scenarios | | |
|  | Note that we measured the tumor with 2D measurements in the axial plane. | | |
|  | See figure 1 of the manuscript. | | |

Commentary

| **Number** | **Question** | **Answers** |
| --- | --- | --- |
| 4.1 | We would be pleased if you gave us feedback on this questionnaire.(optional) |  |
| 4.2 | Would you like to be informed about the results of the study? | Yes   No |
